# In vitro and molecular docking analysis of chalconeimine derivatives with α-glucosidase

**DOI:** 10.6026/97320630016949

**Published:** 2020-11-30

**Authors:** R Asaithambi, C Palanivel

**Affiliations:** 1 PG and Research Dept. of Chemistry, Government Arts College, C. Mutlur, Chidambaram - 608102, Tamilnadu, India

**Keywords:** nanoparticles of TiO_2_ ZnO, green synthesis, diphenyl-picrylhydrazine (DPPH), antioxidant

## Abstract

It is known that α-glucosidase is linked with the antioxidant activity. Therefore, it is of interest to document the in- vitro and molecular docking analysis of chalconeimine derivatives with α-glucosidase (PDB ID: 2ZEO) for further consideration.

## Background

Moieties of hydrazone are very common in naturally occurring compounds and are critical because of their substantial biological effectiveness, which includes anticancer, cytotoxic, anti-malarial, anti-microbial, anti-inflammatory, anti-oxidant and many others [1]. It is well established that the catalyst plays a crucial role for the synthesis of hydrazones along with other favorable conditions [2-5]. Worldwide scientists are always trying to modify the catalyst to improve the reaction efficiency and also to reduce its toxicity [6]. Green chemistry is a chemical process that reduces or removes environmentally hazardous chemicals from the application and production [7]. Organic solvents required to perform some organic reactions are often toxic and costly [8]. Because of this, the removal of these solvents is an acceptable job for nature [9]. Thanks to their greater surface area per unit mass, specific nano catalysts have recently gained considerable attention [10]. Metal oxides are ZnO, CuO, SiO_2_, CeO_2_, Fe_3_O_4_, CaO, In_2_O_3_ ZrO_2_, etc. In nano form, have recently attracted considerable attention as effective, environmentally sustainable, heterogeneous catalysts and have found immense applications in various organic transformations [11]. TiO_2_ and ZnO nanoparticles have been used in various fields, including optoelectronics, ferromagnetism, piezoelectric transducers, solar cells, gas sensors, etc [12,13]. These also possess antibacterial efficiencies and antioxidants. Various nanostructures of TiO_2_ and ZnO, such as nanoparticles, nanorods, nanowires, nanobelts, nanotubes, nanobridges and nanonails, nanowalls, nanohelixes and polyhedral cages, have been synthesized and characterized well in recent years [14,15]. Therefore, in the past decade, tremendous applications of different nano-TiO_2_-and ZnO morphologies have been seen as catalysts in various organic name reactions including Mannich reaction, knoevenagel condensation and various organic transformations [16]. Low toxicity, low corrosion, large surface area, high pores volume, reusability, low cost, environmental sustainability and commercial availability make this Lewis acid heterogeneous nano catalyst superior to others [17,18]. Hydrazone compounds exhibit increased catalytic activity compared to their types of bulk sizes. Another subject of this work is the investigation of the power of the synthesized compounds in terms of antioxidant activity [19,20]. Usually, compounds with antioxidant activity could be eliminated through these compounds because of their reduction properties and chemical structure used as transitional metal chelators and negative effects of free radicals [21]. Continuing our work on the synthesized 4a-e compounds with high yields and evaluate the antioxidant activity, molecular docking studies compared the results with BHT as synthetic antioxidants.

## Materials and Methods:

Zinc acetylacetonate (Z_n_C_10_H_14_O_5_), Titanyl acetylacetonate (C_10_H_14_O_5_T_i_) were reagent of Sigma Aldrich and used as such. All glassware was cleaned acid followed by thoroughly washing with distilled water, deionized water and water is used as a throughout experiment. The melting points of all synthesized compounds were determined by an open capillary method and were uncorrected. Precoated plates (silica gel 60 F254) were used for analytical thin-layer chromatography (TLC) for monitoring the reaction progress, and spots were visualized with iodine chamber. Infrared (IR) spectra (KBr disc) were recorded on a Shimadzu (FTIR-4100) spectrometer. Proton nuclear magnetic resonance (1H NMR) spectra and Carbon nuclear magnetic resonance (13C NMR) spectra were recorded on a Bruker Advance II 400 MHz spectrometer in DMSO-d6. The structural characterization of the prepared nano metaloxide was reported by X-ray diffraction technique on SHIMADZU-6000. The surface morphology was studied by using SEM (JEOL-JES-1600). High-resolution transmission electron microscopy (HR-TEM) images were taken using a JEOL-JEM-2010 UHR instrument operated at an acceleration voltage of 200 kV with a lattice image resolution of 0.14 nm.

## Docking studies:

α-glucosidase ((PDB ID: 2ZEO) was retrieved as a PDB file from the RCSB Protein Data Bank (http:/www.rcsb.org/pdb/). The Auto Dock Tools (ADT) used to prepare protein. Both water molecules and hetero-molecules were removed in protein preparation, the B chain. Polar hydrogen atoms were added, along with Kollman charges. Docking study was conducted to test the binding affinity of synthesized hydrazone derivatives to active α-glucosidase enzyme residues (PDB ID: 2ZEO) using the program, Auto Dock 4.2.6. To get the best conformational docking state, a grid box covering the α-glucosidase active site residues of the target protein was created. The size of the docking grid box was set to 60 ranging from 0.514 Å to 25.19, 25.805, and 46.378 X, Y and Z coordinates. Use of the Lamarckian Genetic Algorithm (LGA) to docking.

## DPPH free radical scavenging activity:

Antioxidant potency of the test samples and standard was tested using 2,2-diphenyl-1-picrylhydrazyl radical (DPPH). In methanol solution (0.2 mM) 3.9 mL of DPPH is added to sample or standard methanol solutions (0.1 mL) at concentrations of (20, 40, 60, 80, 100 µg / ml). Command is 0.1mL of methanol added to DPPH solution 3.9mL. The procedure was done in triplicate. In the dark, the incubation is performed at room temperature for 90-minutes, then the absorption was measured at 517 nm. Control is taken without test substances, but an equivalent amount of methanol taken. We used as normal ascorbic acid. See Table 2(see PDF) for the results obtained from the DPPH assay. The ED50 plus V 1.0 was used to assess the IC50 values for normal and test samples. Inhibition percentage is measured using formula:

## Synthesis of TiO_2_-ZnO nanofilms:

TiO_2_-ZnO thin films were processed on microscopic glass substrates, at a temperature of 400°C using spray pyrolysis technique. For making deposition, 0.1M of Zinc acetylacetonate (ZnC10H14O5) was dissolved in ethanol, together with Titanyl acetylacetonate (C_10_H_14_O_5_T_i_) at the atomic concentration and then dissolved in the deionized water. This synthesized solution was sprayed on the microscopic glass substrates, having the dimension of 75x25 mm^2^ at the temperature of 400°C (Ts=400°C). Before the preparation of thin film, glass substrates were well cleaned with hydrochloric (HCl) solution followed by water bath, acetone, rinsed with distilled water and allowed to dry in oven. Before making deposition, the substrates were pre-heated for specific time and then the synthesized solution was sprayed respectively. When a precursor aerosol droplets moving close to the heated substrate, a pyrolytic decomposition process exists, and as a result, high quality TiO_2_-ZnO films were developed. After that, the deposited films were allowed to cool slowly up to room temperature, followed by washing with distilled water, dried and then annealed at 500°C in air. Finally, the thin films deposited were characterized.

## Structural studies:

**Figure 1:**
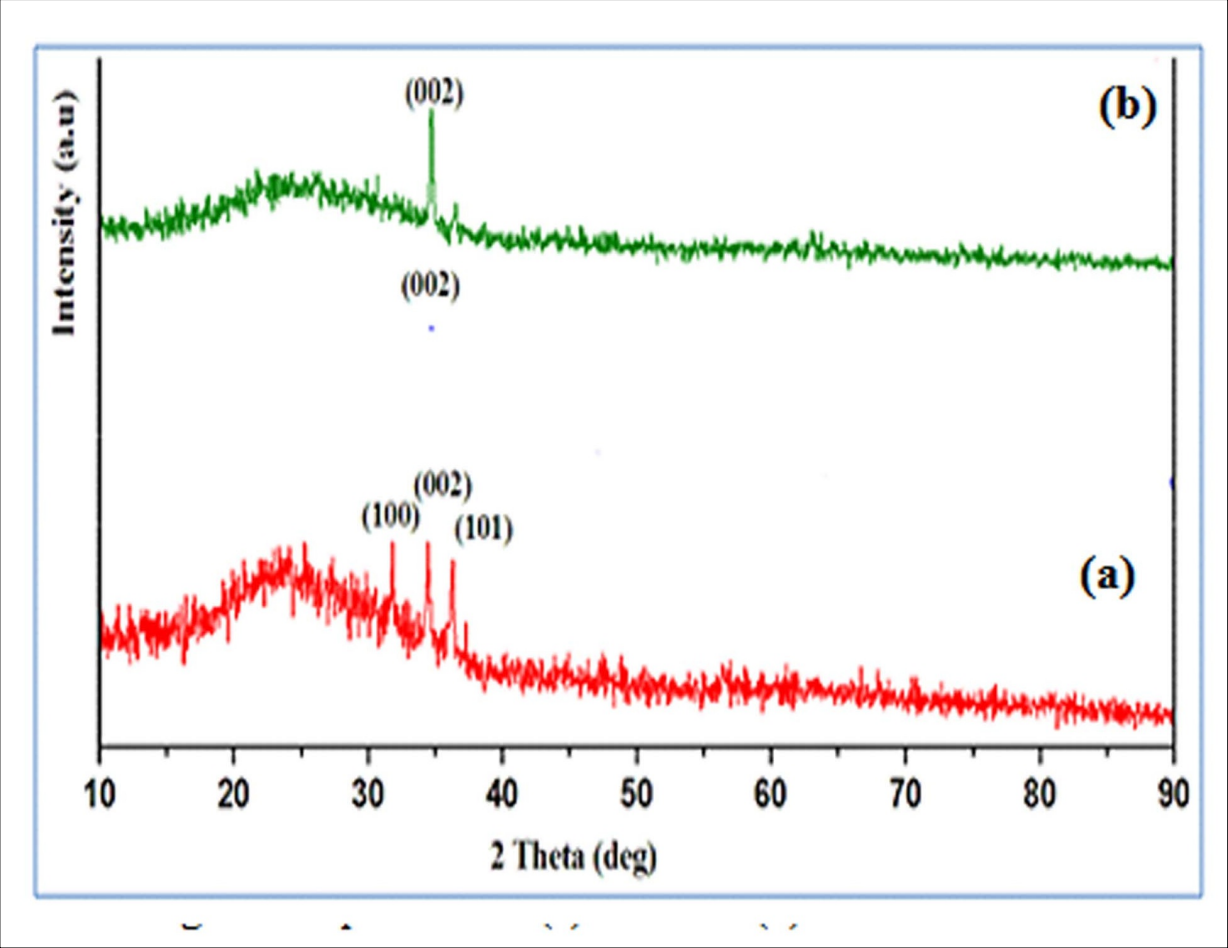
XRD pattern of pure ZnO and TiO_2_-ZnO thin films

Figure 1 revealed that XRD pattern of pure ZnO and TiO_2_-ZnO thin films deposited on glass substrates at 400°C (optimized temperature) All the ZnO: TiO_2_ films are preferentially oriented along (002) plane c - axis with hexagonal wurtzite structure and free from the formation of secondary phases. The diffraction peaks detected for the films corresponding to (101), (002) and (100) planes are the indicative of hexagonal wurtzite structure (JCPDS card No 36 - 1451). No second phase is detected in XRD pattern of pure ZnO and TiO_2_- ZnO. Pure ZnO films shows the presence of sharp peaks with increased intensity comparable to the planes (100), (002) and (101). This is due to the uniform distribution of crystallites in the ZnO sites, because Ti^4+^ ions can replace Zn^2+^ ions in ZnO sites as the ionic radius of Ti^4+^ (0.68 Å) is smaller than that of Zn^2+^ (0.74 Å). The intensity of the plane (0 0 2) is also enhanced TiO_2_ doped ZnO films, while the other planes like (100) and (101) are observed with low intensity. The strengthening of (002) planes is due to the substituted Ti ions at Zn sites into the wurtzite structure, which reduces the surface energy.

## Surface morphology and composition analysis:

**Figure 2:**
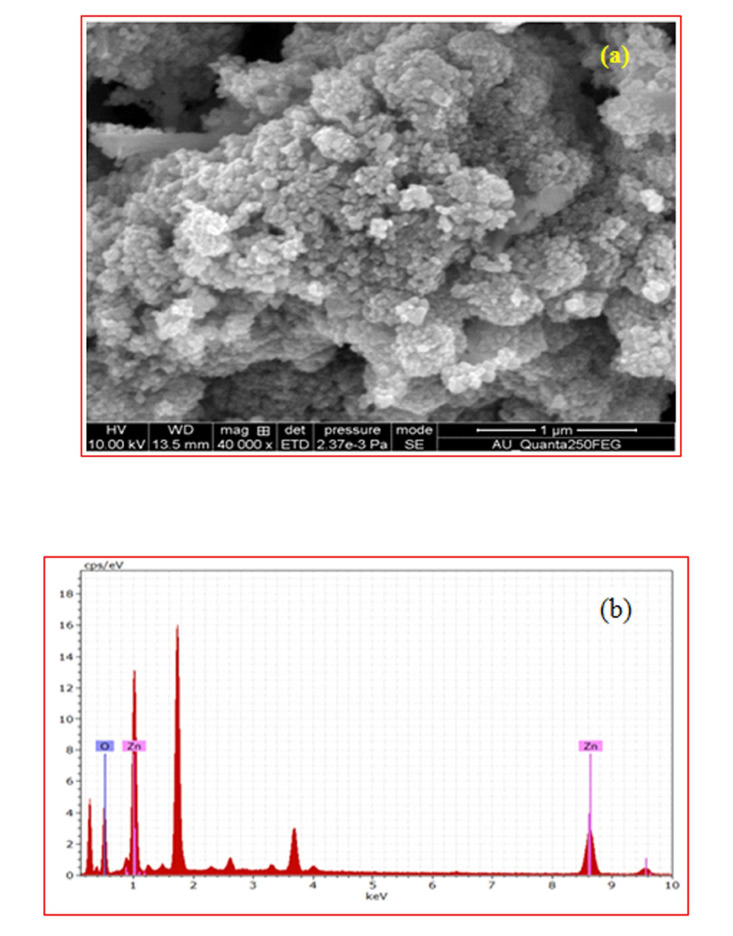
SEM images of (a) ZnO and (b) TiO_2_ -ZnO thin films.

**Figure 3:**
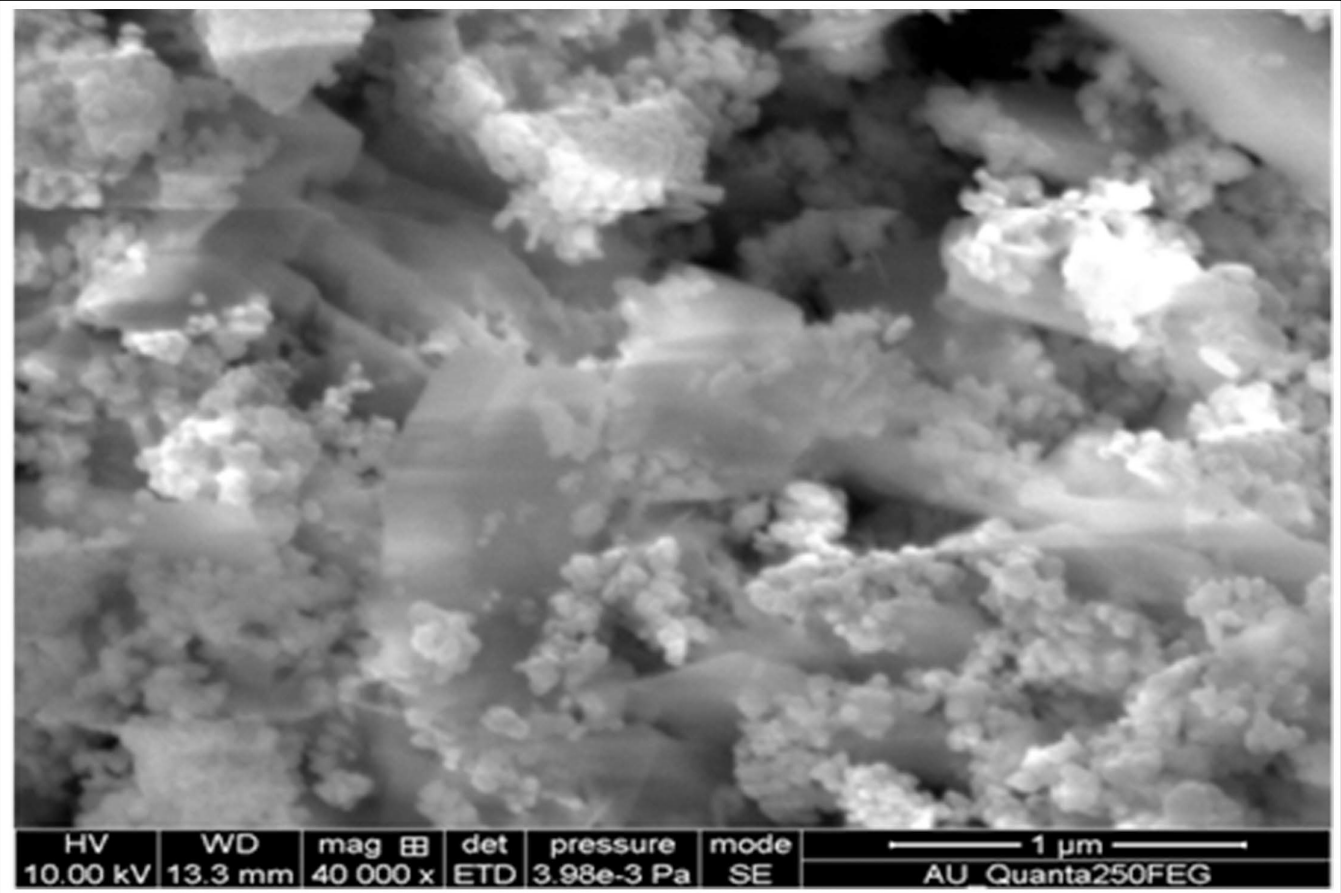
EDX spectrum of (a) Pure ZnO and (b) TiO_2_ -ZnO thin films.

**Figure 4:**
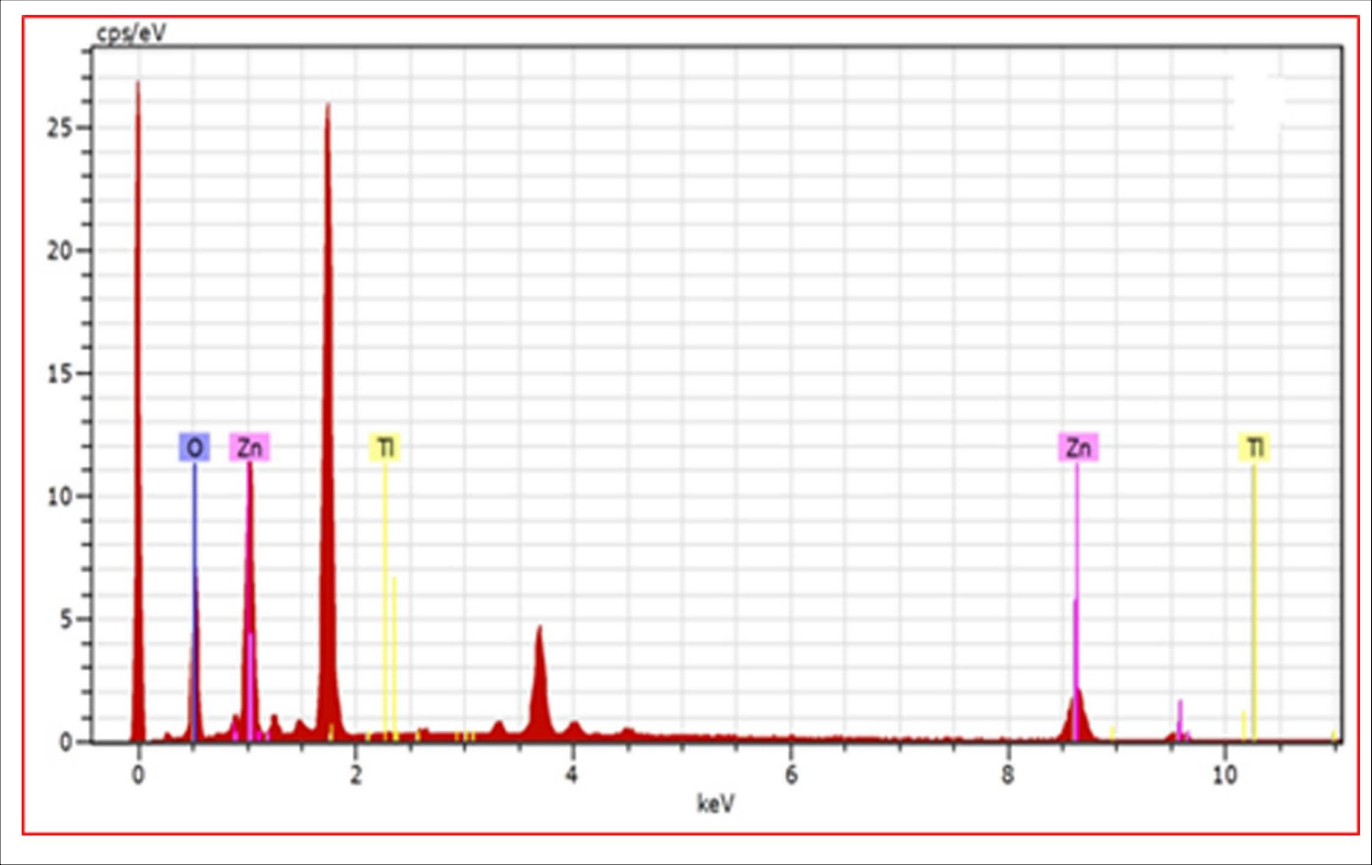
SEM images of (a) TiO_2_ -ZnO thin films.

Figure 2a exhibits the morphology of ZnO and TiO_2_-ZnO films with different atomic percentage deposited at the substrate temperature of 400°C. The pure ZnO film exhibits the dense continuous well coverage of the substrate with nano spherical particles nature is observed in Figure 2b. The elemental analysis of pure ZnO thin films are characterized by EDS spectrum analysis revealed that pure ZnO film confirms the presence of Zn and O observed. Figure 3a.The TiO_2_-ZnO film exhibits the dense continuous well coverage of the substrate with nano nano sheet shaped is observed. The Figure 4 TiO_2_-ZnO shows film confirms the presence of Ti along with Zn and O with respectively.

## HR-TEM-analysis:

**Figure 5:**
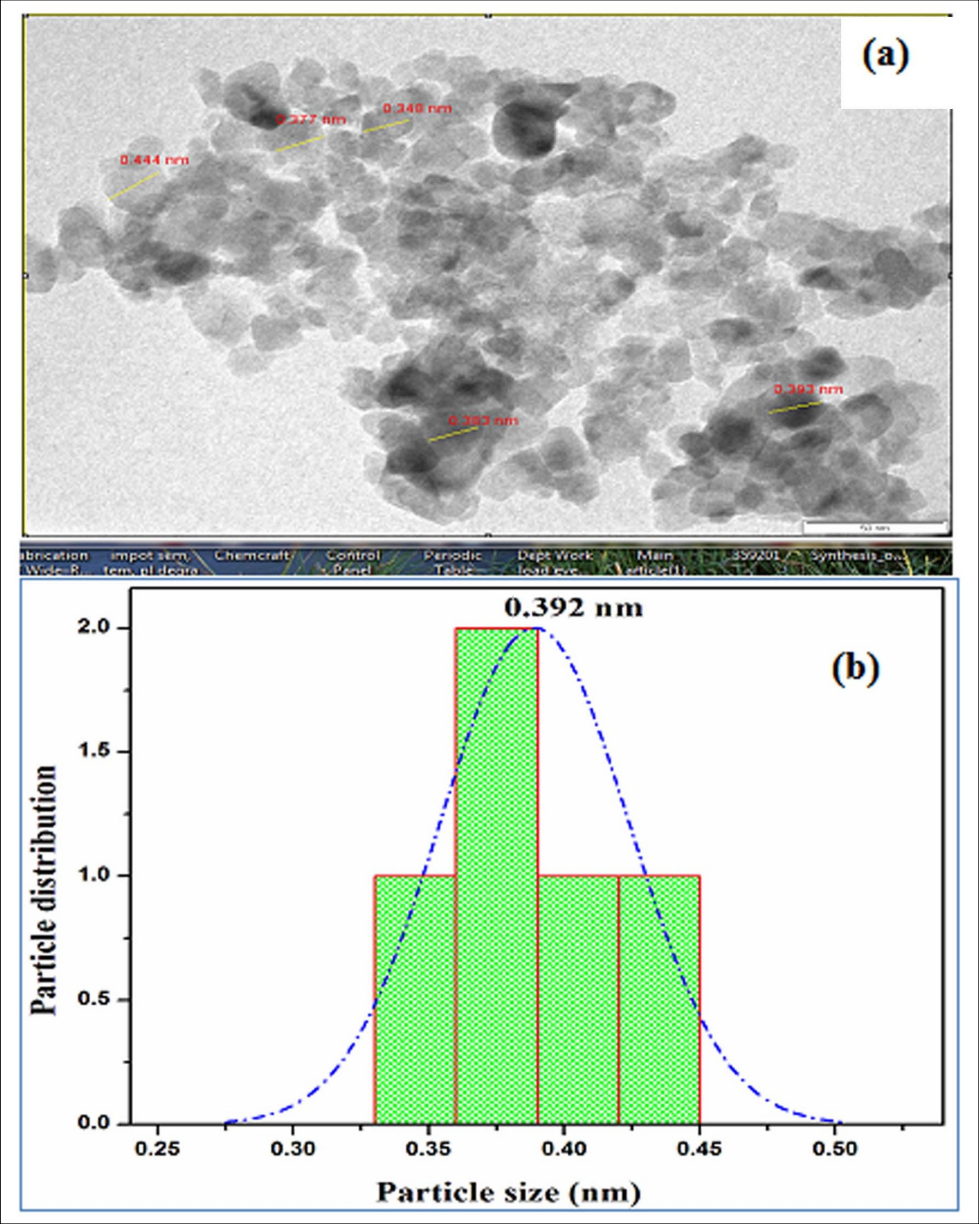
HRTEM (a) images of TiO_2_ -ZnO thin film (b) Avarage particle size in selected area highlighted by TiO_2_ -ZnO particle

**Figure 6:**
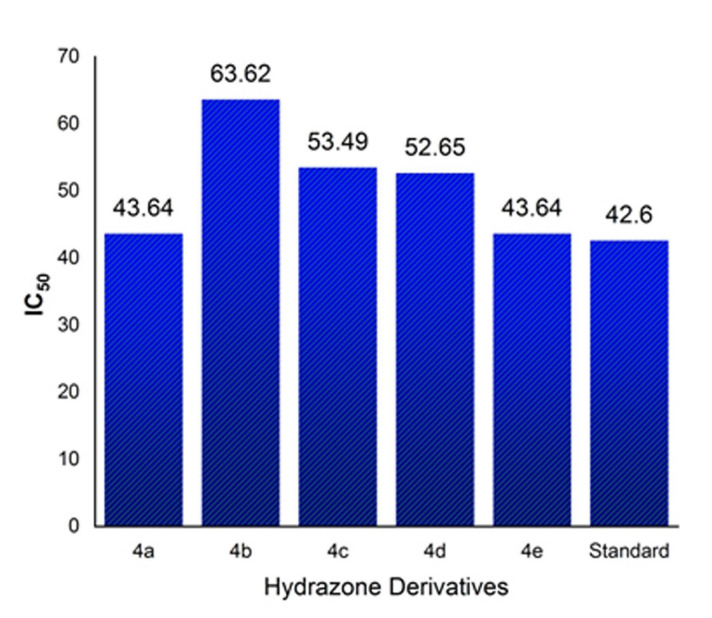
Antioxidant efficacy of synthesized hydrazone derivatives by DPPH method

Figure 5a. HRTEM analysis of TiO_2_-ZnO films. It is seen that the crystal grain size, crystalline volume, and crystallinity reduced as the doping ratio increased in accordance with the XRD results. The tiny spherical particles are having high surface area and making superior contact area with microbe, which can be extremely responsible for better solar cells. Figure 5b shows the particle size distribution in range 0.392 nm selected particle area highlighted Figure 6a respectively.

## Results and Discussion:

In our present work, a series of chalconeimine derivatives of (E)-3-(4-(difluoromethoxy)-3-hydroxyphenyl)-1-(2,4-dimethylthiazol-5-yl)prop-2-en-1-one (3) and various substituted hydrazide were synthesized by nucleophilic addition of hydrazide to substituted aromatic chalcone under reflux in ethanol in the absence of catalyst. The reaction took an extended period of time (6-8 hours) to complete with a modest yield of the products (60-72 %) (Table 2 - see PDF). To establish an eco-friendly approach for the synthesis of biologically active chalconeimine derivatives, we explored the efficacy of the TiO_2_-ZnO nanocatalyst by performing the reaction of (E)-3-(4-(difluoromethoxy)-3-hydroxyphenyl)-1-(2,4-dimethylthiazol-5-yl) prop-2-en-1-one (3) and various substituted hydrazide in (1:1) molar ratio. Throughout our experiments, we investigated the optimum reaction conditions for solvent quality, reaction temperature and catalyst quantity on a model reaction using (E)-3-(4-(difluoromethoxy)-3-hydroxyphenyl)-1-(2,4-dimethylthiazol-5-yl) prop-2-en-1-one (0,01 mol) and furan-2-carbohydrazide to determine the best conditions.

The model reaction was investigated for various concentrations 5, 10, 15, 20 and 25 mol % of the TiO_2_-ZnO nanocatalyst at reflux temperature ethanol solvent condition to achieve the optimum concentration of the catalyst. From results, it is apparent that 20 mol% of the catalyst is sufficient to achieve optimum yield in the shortest reaction time. Using less than 20 mol % of the catalyst, the product's moderate yield (76-82 %) was achieved with extended reaction times, while the catalyst yield was not further increased with an excess mol% (25 mol %), probably due to the saturation of the catalyst's catalytic sites (Table 1 - see PDF). The model reaction was carried out in different solvent systems for the study of the solvent effect. The model reaction was first investigated in MeOH and Water the reaction took longer (4 and 10 hrs) with moderate yields of 70% and 56% respectively, while in DMF the sample was obtained in better yield (69%) after refluxing for 5 hrs. In fact, there was a notable increase in product yield in a shorter period of time when the model reaction was conducted under ethanol solvent. Considering the above findings, it was concluded that the best reaction condition for the synthesis of present chalconeimine derivatives in excellent yields is ethanol solvent. The efficacy of this method was investigated for the synthesis of chalconeimine derivatives (Scheme 1) using these optimized reaction conditions discussed above, and the results obtained are presented in Table 2(see PDF). The reusability of the catalyst was also explored for the selected model reaction. The catalyst was reused five times and the results demonstrate that the catalyst can be reused without a significant reduction in the yield (Table 3 - see PDF). DPPH radical scavenging ability of some synthesized compounds was evaluated and compared with standard antioxidant BHT.

**Figure 7:**
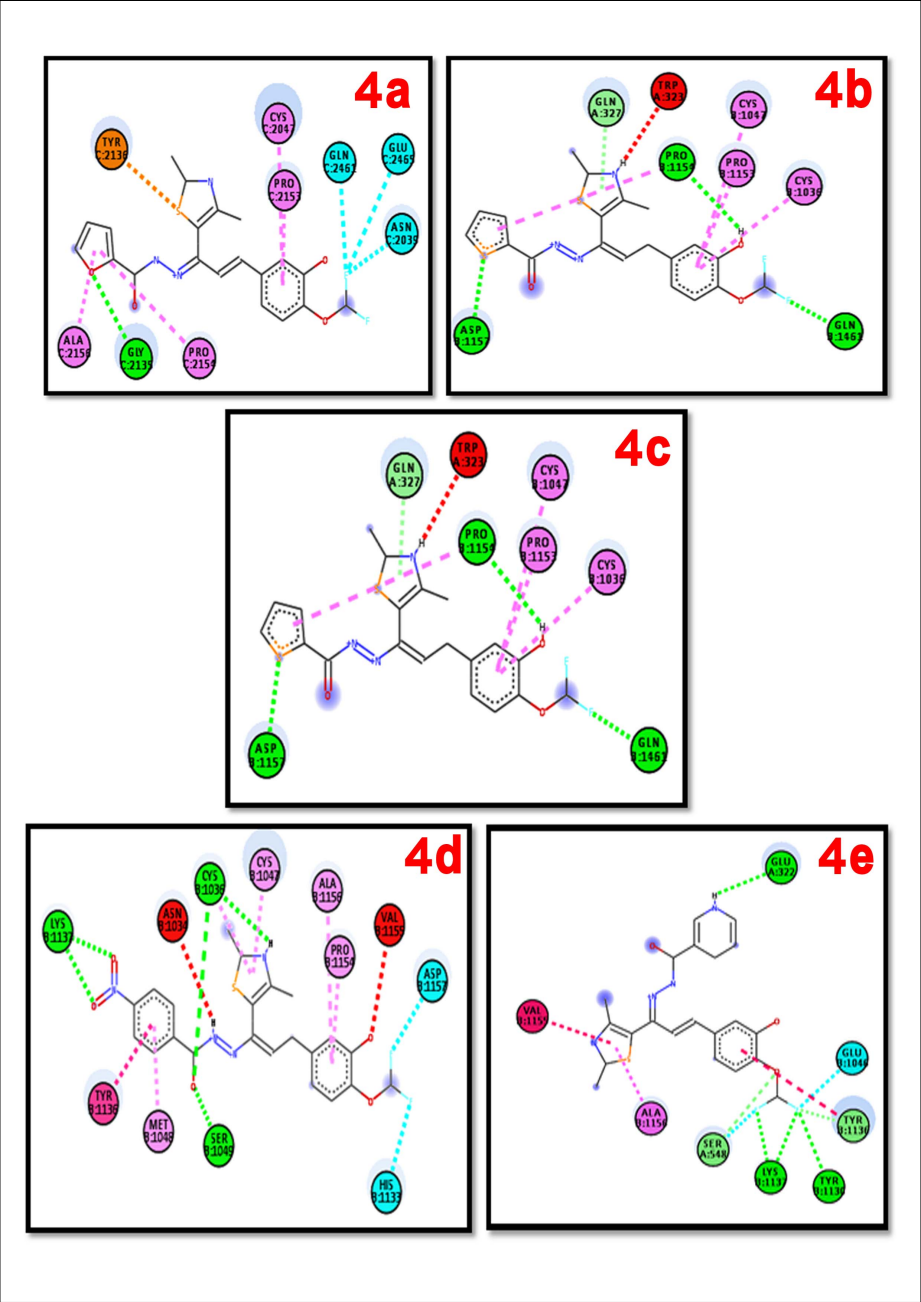
2D pictorial representation of various intraction of hydrazone with 2ZEO protein receptor

The graphical representation of antioxidant activity inhibition concentrations (IC50) of compounds 4a-e was shown in Figure 6,Figure 7. The perusal of Table 4(see PDF) revealed that compounds 4a-e possessed moderate to good antioxidant activity (Figure 3). Although 4a IC50 = 43.64 µg/mL and 4e IC50 = 43.68 µg/mL showed good antioxidant activity. Similarly, the active compounds 4d IC50 = 52.65 µg/mL and 4c IC50 = 53.49 µg/mL exhibited moderate radical scavenging ability at the concentration of 100 µg/mL which may result due to the presence of furan oxygen, nitro, sulphonyl and nicotinyl groups respectively in that compounds. The result of the docking studies has been carried between the human antioxidant enzyme receptor (PDB: 2ZEO) and the complexes are reported in Table 5 (see PDF). The complexes exhibited comparable binding interaction energy values in the in the range of -10.1 to - 11.5 kcal mol-1. The complexes 4c, 4d and 4e showed more negative binding values i.e -11.3, -11.0 and -11.5 kcal mol-1 respectively when compared with the standard butylated hydroxytoluene -10.5 kcal mol-1. The stability of the synthesized derivative 4d and 4e were evaluated by determining the number of hydrogen bonding interactions between amino acids of the protein molecules with the synthesized chalconeimine derivatives. The result indicates that the complex 4d and 4e having the highest number of hydrogen bond intraction ie 5 and also has greater binding affinity with the human antioxidant enzyme active site residues. Hydrogen bond interactions of compound 4a forms with the active site enzyme residue of GLY 213, GLN 246, GLU 246 and ASN 207 through the oxygen atom of furan ring and with two fluorine atom of compound 4a, and other interactions involved pi -alkyl interactions with ALA 215, PRO 215, CYS 204. Further,π -sulfur interactions of thiazole ring of compound 4a with TYR 213 of active site amino acid residues respectively and compound 4b forms hydrogen bond interactions with amino acid residue of ASP 115,GLN 146 and PRO 115 with sulphur, fluorine and hydroxyl group of compound 4b & pi-alkyl interaction with PRO 115, CYS 103 and CYS 104 with its benzene ring. At the binding site of α-glucosidase enzyme sulphur, fluorine and oxygen atom of compound 4c formed hydrogen bond intraction with ASP 115, GLN 146 and PRO 115 and other interactions π-alkyl with CYS 104 and PRO 115. Compound 4d, two oxygen atom of nitro group and one oxygen atoms of - C=O group have connected with LYS 113, SER 104 and CYS 103 by four Hydrogen bond interactions and benzene ring forms five pi-alkyl interactions with ALA 115, PRO 115, CYS 104, MET 104 and TYR 113. Hydrogen bond interactions of 4e formed through fluorine and oxygen atom with hydrogen atom of LYS 113, TYR 113 and GLU 322. Further, π alkyl interaction through ALA 115 and VAL 115 r of protein and thiazole ring of compound 4e.

## Conclusion

Document the in-vitro and molecular docking analysis of chalconeimine derivatives with α-glucosidase (PDB ID: 2ZEO) for further consideration.

## References

[1] Vasava MS (2020). Mini Reviews in Medicinal Chemistry..

[2] Kobayashi S (1999). Chemical Reviews..

[3] Quasdorf KW (2014). Nature..

[4] Kalinowski DS (2008). Journal of medicinal chemistry..

[5] Xia Y (2017). Chemical reviews..

[6] Banerjee B (2017). Ultrasonics sonochemistry..

[7] Poliakoff M (2002). Science..

[8] Alcalde M (2006). TRENDS in Biotechnology..

[9] Visser AE (2000). Industrial & Engineering Chemistry Research..

[10] Koenigsmann C (2011). Journal of the American Chemical Society..

[12] Kumar V (2019). Biotech..

[13] Sengwa RJ (2019). Journal of Materials Science: Materials in Electronics..

[14] Chen F (2009). Biotechnology and bioengineering..

[15] Noori S (2018). Food control..

[16] Hosseini-Sarvari M (2013). Current Organic Synthesis..

[17] Mardhiah HH (2017). Renewable and sustainable energy reviews..

[18] Liu F (2018). ACS Catalysis..

[19] Sherer M (1997). Interdisciplinary International Journal of the American Cancer Society..

[20] Nabatipour S (2020). Journal of Molecular Structure..

[21] Pham-Huy LA (2008). IJBS..

